# Assessment of Wettability and Contact Angle of Bonding Agent with Enamel Surface Etched by Five Commercially Available Etchants: An In Vitro Study

**DOI:** 10.1155/2021/9457553

**Published:** 2021-10-08

**Authors:** Deepika Katyal, Aravind Kumar Subramanian, Adith Venugopal, Anand Marya

**Affiliations:** ^1^Department of Orthodontics, Saveetha Dental College, Saveetha Institute of Medical and Technical Sciences, Chennai 600077, India; ^2^Department of Orthodontics, Faculty of Dentistry, University of Puthisastra, Phnom Penh 12211, Cambodia

## Abstract

**Background:**

The quantity and quality of the etching pattern produced by acids play a significant role in the wettability and contact angle of the adhesive with the enamel surface in orthodontics. The better the etch pattern, the more the surface energy of the enamel, better the penetration of the adhesive, which ultimately results in better bond strength. The present study aimed to evaluate the contact angle of the bonding agent with the enamel surface etched by five commercially available etchants and check if any difference existed between the five.

**Materials and Methods:**

Twenty-five human maxillary and mandibular central incisors and premolars extracted for orthodontic or dental purposes were used in this study as samples. The teeth were allocated into five groups based upon the etchant used to etch the enamel surface. After the samples were etched, a hard tissue microtome was used to create thin slices of the enamel surface. The samples were then exposed to bonding agent Ormco Enlight. The bonding agent was released in the form of droplets onto the enamel slices mounted on a contact angle goniometer. The contact angle values were tabulated, and statistical analysis using the one-way ANOVA test was carried out.

**Results:**

The contact angle measurements of the etchant group, DPI, were the lowest, while D-tech has the highest contact angle values. However, statistical analysis revealed no statistically significant difference between the contact angle measurements of the five groups included in the study.

**Conclusion:**

No statistically significant difference existed between the five etchant groups included in the study. However, commercially available etchant DPI showed lesser contact angle and thus better wettability in comparison to other groups. Further elemental analysis and surface analysis are required to validate these results.

## 1. Introduction

Enamel conditioning by an etchant results in microporosities on the tooth surface. The creation of microporosities also increases the enamel surface's surface energy [1]. The increase in surface energy of enamel permits the penetration of the bonding agent into the enamel. The formation of enamel tags, which produce mechanical retention of the bonding agent, is also influenced by the quantity and quality of the etchant used [2, 3].

The quantity and quality of the etching pattern produced by acids play a significant role in the wettability and contact angle of the adhesive with the enamel surface in orthodontics. The better the etch pattern, the more the surface energy of the enamel, the better the penetration of the adhesive, which ultimately results in better bond strength [4]. Bracket bonding to the enamel surface should result in a strong enough attachment to endure the forces of mastication and orthodontic treatment without dislodging; also, at the same time, there should be minimal damage to the surface during debonding [5]. The efficiency of a bonding agent depends upon its bond strength and debonding character–two factors that are affected by the penetration of the orthodontic adhesive.

Many studies have been performed in the past comparing the etch patterns with different acids and etching times, with different dentin adhesives [6]. The depth of resin penetration into the enamel with different etching methods has also been evaluated in the past [7, 8]. There is also a lot of literature comparing the self-etching primers with the conventional two-step etching process involved in orthodontic bonding [6–10]. Various studies are also available comparing the shear bond strength of orthodontic composite using different etchants and adhesive systems [11]. Most of the studies primarily aimed at evaluating the influence of different etchants, varying etching times, and different orthodontic adhesives on the “strength of bonding” [12]. Also, the “pattern of etching” with different etchant and adhesives has remained the prime focus of interest [4, 13]. However, no study is available that compares the influence of commercially available and commonly used etchant systems on the wettability of the orthodontic bonding agent with the enamel surface. Does a relationship exist between the type of etchant used and its influence on the “wetting” efficiency of the bonding agent onto the enamel surface remains an area of interest. Wetting is commonly characterized by the contact angle, which is defined as the angle between the tangent to the liquid-vapor interface and the solid surface at the three-phase contact line [14].

Ormco Enlight (Glendora, California, USA, 007058) is a commonly used bonding agent used in orthodontic practice, while Prime manufactured by Prime Dental Products (Thane, Maharashtra), ANABOND manufactured by Anabond Stedman Pharma Research (Chennai, Tamil Nadu), D-tech manufactured by D-tech Orthodontics (Pune, Maharashtra), and DPI manufactured by DPI (Mumbai, Maharashtra) are some of the common and economical options of etchants available in India. Ivoclar manufactured by Ivoclar Vivadent (United States of America) is a comparatively less economical and less commonly used etchant.

This study aimed to evaluate the wettability and contact angle of the commonly used orthodontic bonding agent (Enlight) when used with five different commercially available etchants mentioned above and analyze if any difference was present in the contact angle of the bonding agent with the etched enamel surface.

## 2. Materials and Methods

### 2.1. Study Setting

This study was performed in a university setting at Saveetha Dental College and Hospitals, Chennai. The ethical committee granted the ethical approval for this study at the university. The study was carried out in vitro.

### 2.2. Sample Size Calculation

The sample size and calculation were carried out from a previous study performed by Pawan Kumar Bhandari [1]. A power value of *P* = 95 was calculated, and the sample size was calculated to be *N* = 25, with 5 groups of etchants and 5 samples in each group.

The samples were randomly allocated into the 5 groups of etchants by the simple random sampling technique.

### 2.3. Sample Preparation

Twenty-five human maxillary and mandibular central incisors and premolars, extracted for orthodontic or dental purposes, were used in this study as samples. The teeth were first visually examined to be devoid of caries, enamel cracks, fluorosis, or abrasion. The samples that were found to be free of caries and restorations and were stored washed with water after extraction and stored in 0.1% thymol solution to prevent dehydration and bacterial growth. The teeth were allocated into five groups having five teeth in each group. Each tooth was mounted vertically in a self-cure acrylic resin, so that the crowns were exposed.

In group A, the enamel surfaces were conditioned with 37% phosphoric acid–Prime, manufactured by Prime Dental Products (Thane, Maharashtra) for 30 seconds, rinsed thoroughly under running water for another 30 seconds, and dried with compressed oil-free air for 5 seconds until a frosted whitish appearance was seen on the enamel surface. Similarly, in group B, the enamel surfaces were conditioned with the etchant Anabond manufactured by Anabond Stedman Pharma Research (Chennai, Tamil Nadu). In group C, D-tech was manufactured by D-tech Orthodontics (Pune, Maharashtra), group *D* with DPI manufactured by DPI (Mumbai, Maharashtra), and group *E* with Ivoclar manufactured by Ivoclar Vivadent (the United States of America).

**Figure 1:**
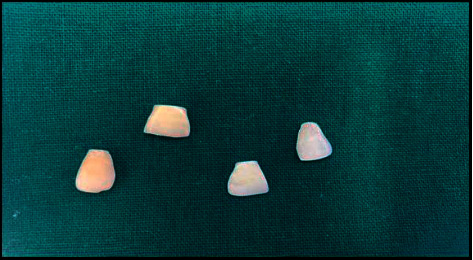
The sample teeth reduced to enamel discs using microtome.

**Figure 2:**
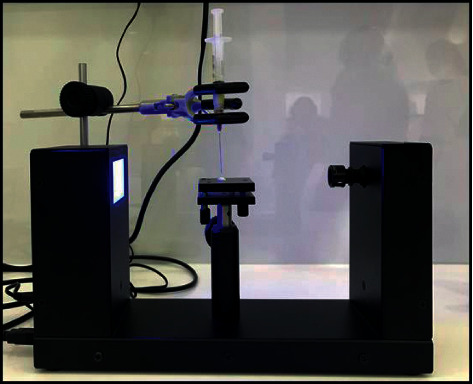
A contact angle goniometer used to measure the contact angle.

**Figure 3:**
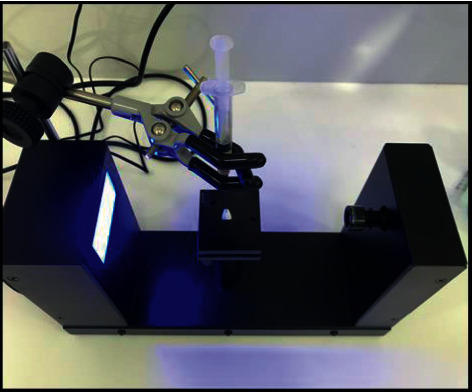
Mounting of enamel disc and bonding agent.

A hard tissue microtome was used to create thin slices of the enamel surface (Figure 1). The samples were then exposed to the bonding agent Ormco Enlight. The bonding agent was released in droplets onto the enamel slices mounted on a contact angle goniometer (Figures 2 and 3).

### 2.4. Wettability and Contact Angle

**Figure 4:**
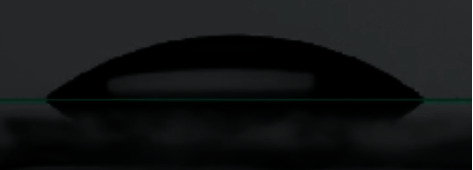
Baseline positioning using droplet reflection.

Measurement of the static contact angle was performed using the sessile drop method by placing a drop of liquid adhesive in a volume of 1.0 *µ*L using a micropipette. A set of 3 images was captured within 2 s after placing a drop of liquid adhesive on the enamel specimen, and subsequent contact angle was measured by axisymmetric drop shape analysis using the Ossila contact angle goniometer. The first step in the measurement is to obtain an image of a droplet on a flat surface. Once an image of the droplet on the flattened specimen is obtained, the baseline of the droplet is manually marked at the interface of the real image and its reflection (Figure 4).

**Figure 5:**
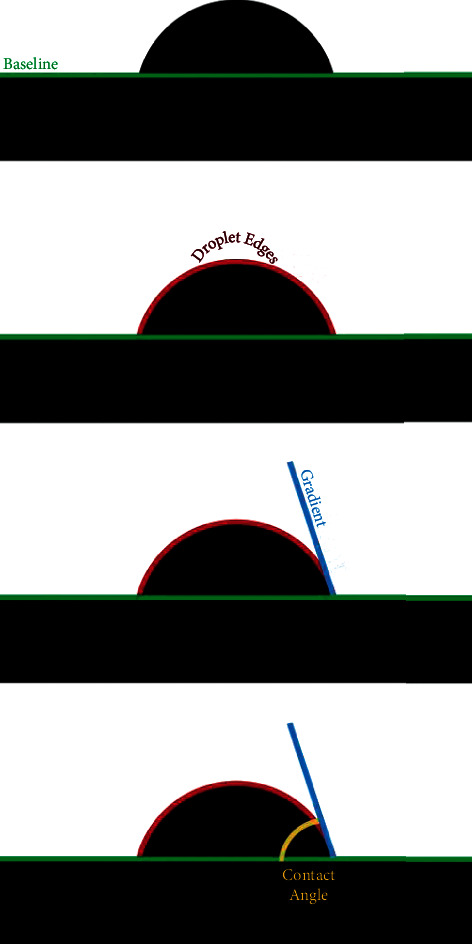
Stages of contact angle analysis.

**Figure 6:**
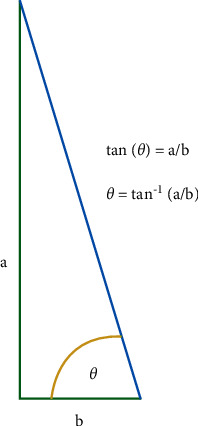
Measurement of contact from the gradient of the tangent of the slope to the baseline.

**Figure 7:**
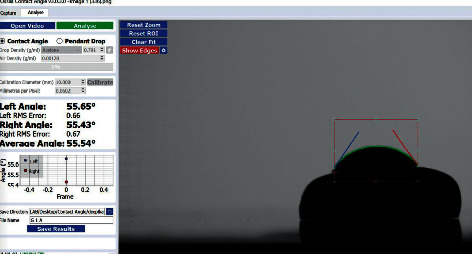
Contact angle measurement of the included samples.

The tracing of the droplet edge and the gradient of the tangent of the droplet edge to the point where it meets the baseline is programmatically marked by the contact angle goniometer software, and the contact angle between them is calculated on the left and right sides of the sample (Figures 5–7). A single operator was involved in marking the baseline of the droplet within the field of interest for all the samples, and their contact angle was measured. 2 samples were randomly selected from each group (*N* = 10), which were washed, reetched, and air-dried. The sessile drop contact angle measurement was repeated for these samples by the same observer.

### 2.5. Statistical Analysis

The data for each group were calculated and tabulated in Excel. It was later exported to SPSS (version 23) for statistical analysis. The descriptive statistics for each group were carried out using SPSS software (Table 1). The Shapiro–Wilk test was used to assess the normality of the data.

The one-way ANOVA test was carried out between five independent variables, the etchant groups, and the dependent variables and was the contact angle of enamel with the bonding agent. Kappa statistics was done to assess the intraexaminer reliability of the results.

## 3. Results

**Figure 8:**
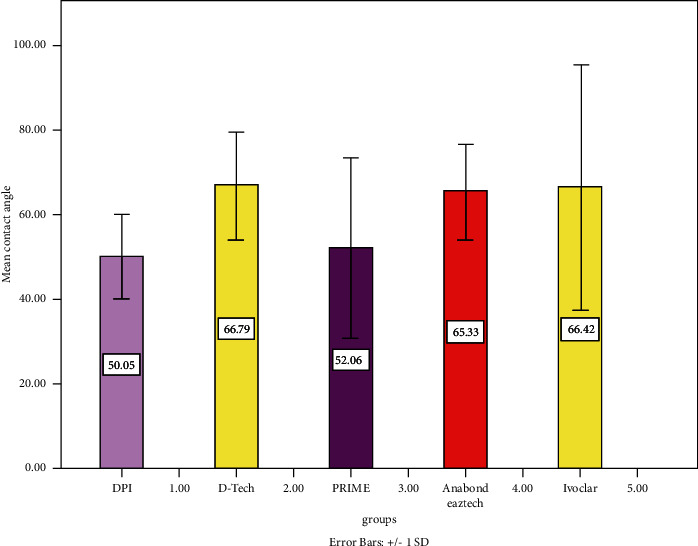
Bar graph indicating the contact angle of the samples in each group.

The descriptive statistics of the samples are described in Figure 8. A one-way ANOVA test was carried out, and the descriptives are represented in Table 2.

The mean contact angle of all groups included in this study was (60.0 ± 17.99). The results of the intergroup analysis revealed no significant difference with *P* > 0.005. The intragroup analysis results revealed no significant difference with *P* > 0.005. Cohen's kappa coefficient value was found to be 0.083, which indicated a substantial level of intraobserver agreement.

The lowest contact angle and better wettability were reported by DPI etchant and the highest by D-tech.

## 4. Discussion

Bonding of orthodontic brackets to the enamel surface requires the creation of microretention in the enamel. The retention quality can be determined by the degree of surface irregularities on the resin enamel interface. The thickness of the smear layer also has a greater effect on the surface wettability [15]. Therefore, applying an acid etchant before the placement of an adhesive bonding system improved adhesion capability [16]. Toledano et al. in their study also reported that the wettability of adhesive improves after acid etching [17]. Etching results in enamel microporosities, increasing the surface energy and creation of resin tags. Increased quality of etchant will thus result in better penetration of the primer into the enamel and eventually result in better bond strength of orthodontic brackets [18].

Various studies have been performed in the past where different methods of acid etching have been compared on account of various parameters such as depth of resin tag penetration, shear bond strength of orthodontic brackets, and enamel characteristics. Studies have reported that 37% phosphoric acid resulted in a greater depth of resin penetration than other etching methods [1]. 37% phosphoric acid concentration with a 30-second application time is the gold standard for enamel etching [19]. Similar findings were reported by Gardner et al. in their study, which indicated the use of 37% phosphoric acid with an optimum application time of 30 seconds [4].

The adaptation and spreading of the liquid adhesive system determine the adhesion [20]. The wetting behavior of dentin bonding agents is influenced by the adhesive blend's physicochemical characteristics, which is influenced by the acid etchant in use. Similar results were obtained in a study performed by Trzcionka et al., which suggested that overdried surfaces exhibit bonding more strongly than overdamp surfaces due to dilution of material [21]. This forms the basis of double-step etching and priming procedures in orthodontic bonding.

Various studies are available comparing various resin adhesive systems and their shear bond strength and microleakage as well as the effect of enamel wetness on bonding agents [22–24]. Bertoz et al. in their study concluded that the best condition for the application of primers to dental enamel occurs in the absence of moisture [25]. A significant difference has also been found between the shear bond strength and the surface roughness of enamel, highlighting the advantage of acid-etched primers over self-etched primers [26].

Although an acid-etched surface will aid in better wetting the enamel surface with the primer, no literature is available on the impact of different commercially available and commonly on use of orthodontic etchants, all containing 37% phosphoric acid, on the orthodontic primer in a two-step etching process.The five groups of etchants and the common primer included in this study are the ones that are commonly in use in clinical practice, easily procurable, and economical.

However, in spite of all the five etchants groups claiming to contain 37% of phosphoric acid, differences exist in the fact that a few groups are more expensive and less economical than the others.

The orthodontic primer is included in this study. Ormco Enlight is also one commonly used in everyday clinical practice.

The contact angle is an indicator of the wetting behavior of any liquid. The angle is formed at a 3-phase interface where solid, liquid, and gas intersect [27]. If the contact angle is less than 90 degrees, the liquid, which in this study is the primer, wets the substrate (etched enamel surface). An angle of more than 90 degrees indicates nonwetting of the surface. A zero-degree contact angle indicates complete wetting. Therefore, contact angle and wettability are inversely proportional to each other, meaning which, the lower the contact angle, the better will be the wettability and vice versa.

The contact angle can be measured by the sessile drop technique or captive bubble technique [28]. In this study, the sessile drop method was used, wherein the contact angle of a liquid on a solid surface can be maintained in a dry environment. All measurements were done using a controlled volume of the primer as any volumetric changes could affect the contact angle (0.1 ml).

The results of this study indicated that no statistically significant difference existed in the wettability and contact angle of the primer with the enamel surface etched by five different groups. Intergroup comparisons also revealed no statistically significant difference.

However, it was interesting that enamel etched with DPI etchant showed the least contact angle of around (50.0 ± 9.96) followed by Prime (52.0 ± 21.4). The etchant is most commonly preferred and less economical than the other groups. Ivoclar showed a contact angle of around (66.4 ± 28.5). These results indicate that similar results could be achieved in terms of orthodontic bond strength with economical options of etchants as well.

Despite 37% phosphoric acid being the chief component of all the five groups of etchants included, the lesser contact angle, and thus, better wettability shown by DPI could be attributed to the lesser viscosity of the etchant in comparison to the other four groups. Studies have shown that a liquid and thinner gel produced a more even etch pattern than thicker gels [29].

However, a definitive cause for this difference in the mean contact angle could be explained by elemental qualitative and quantitative analyses of the etchants in the future, followed by an SEM analysis of their etching pattern.

In orthodontics, self-etching primers, which do not need etching, have also been introduced for bracket bonding [30] and splinting purposes [31]. It could be interesting in the future to test and compare the wettability angle also for these emerging materials.

### 4.1. Limitations

The results of this study should be adapted clinically with caution due to certain limitations associated with the study such as less sample size and less inclusive sample size as only incisors and premolars were included in this study for better preparation of flat enamel discs, which could not have been possible with molar samples.

There is scope for similar studies being performed on more inclusive and larger samples or in vivo in the future. The differences in the composition and mechanism of action of the five group of etchants included in this study require surface and elemental analyses beyond the scope of this study, and the researchers would like to continue research on this aspect in the future.

## 5. Conclusion

No statistically significant difference existed between the five etchant groups included in the study (*P* > 0.005). However, commercially available etchant DPI showed a lesser contact angle (50.0 ± 9.96) and thus better wettability than other groups. However, further elemental analysis and surface analysis are required to validate these results. The results of this study provide scope for further material and elemental analyses of orthodontic etchants to aid in making an informed decision to select the most potent and cost-effective etchant for clinical practice.

## Figures and Tables

**Table 1:** Mean and standard deviation of the contact angle for each group. DPI has the least contact angle followed by Prime, while Ivoclar had the highest.

Etchants included in study	Contact angle	
	Mean	Standard deviation
DPI	50.0500	9.6127
D-tech	66.7850	12.72897
Prime	52.0625	21.42078
Anabond eazetch	65.3250	11.29694
Ivoclar	66.4225	28.85353

**Table 2:** *P* value was found to be more than 0.005, indicating a nonsignificant difference. *P* > 0.005.

One-way anova test	Sum of squares	df	Mean square	*F*	Sig.
Between groups	1110.254	4	277.564	0.826	0.529

## Data Availability

The data used to support the findings of this study are available from the corresponding author upon request.
